# Bullying and Harassment in Downsized Workplaces: What Can We Learn from the 2008 Icelandic Economic Collapse?

**DOI:** 10.3390/ijerph17197180

**Published:** 2020-09-30

**Authors:** Hjordis Sigursteinsdottir, Gudbjorg Linda Rafnsdottir, Gudbjorg Andrea Jonsdottir

**Affiliations:** 1 School of Business and Science, University of Akureyri, Nordurslod 2, 600 Akureyri, Iceland; 2 Faculty of Social and Human Science, University of Iceland, Saemundargotu 2, 102 Reykjavik, Iceland; glr@hi.is; 3 Social Science Research Institute, University of Iceland, Saemundargotu 2, 102 Reykjavik, Iceland; gudbjorg@hi.is

**Keywords:** bullying, downsizing, economic crisis, harassment, job demand, role conflict, social support

## Abstract

Research shows that bullying is a significant workplace issue. A previous study showed increased sickness-related absences among municipality employees during the Icelandic economic crisis in 2008. This led to the following research questions: has bullying and/or harassment increased between the time points of the study up to seven years after the crisis? Did bullying and/or harassment change depending on downsizing? Are quantitative job demands, role conflicts and social support connected to bullying and/or harassment at work and if so, how? The study is based on a four-wave longitudinal balanced panel dataset consisting of those who work within the education and care services operated by Icelandic municipalities. It was seen that bullying and harassment had increased between the time points of the study. Furthermore, employees in downsized workplaces, workplaces with higher quantitative job demands, more role conflicts and less support were more likely to experience bullying and/or harassment than employees in other workplaces. Since the effects may prevail for several years, the study demonstrates that the consequences of downsizing need to be carefully considered and that managers must be supported in that role. As economic crises tend to occur periodically, presently due to COVID-19, the knowledge is both of theoretical and practical importance.

## 1. Introduction

A growing body of social science literature shows that bullying and harassment are significant workplace issues. Regardless of the cause, the consequences of bullying can be severe, including physical and psychological symptoms and negative work-related outcomes [1]. Two common explanations for workplace bullying relate either to the personality of the bullied individual or to the aspects of the work environment. Hutchinson [2] argued that theorizations and policy definitions often emphasize the individual aspects of bullying and overlook the significance of organizational, employment and cultural factors. He also argued that the capacity of policies to prevent or resolve the problem is limited. Hoel, Cooper and Einarsen [3] also highlighted that the economic cost of bullying has received little attention which is somewhat surprising given the magnitude of the problem [4], and the effect of bullying on the individuals targeted [5,6]. This would undoubtedly represent a substantial cost to the organization, whether through payouts to settle claims of bullying and harassment or affecting levels of absenteeism, turnover and productivity as well as team and group performance. Nevertheless, in one of the first studies of workplace bullying, Einarsen, Raknes and Matthiesen [7] focused on psychosocial factors at work as predictors of bullying. Since then, several studies have demonstrated the importance of organizational factors in preventing bullying and other unwanted harassment at work. They revealed that work-related factors such as heavy workload, lack of job security [8,9,10], high job demands, low job control [11,12], role conflict [7,13,14], lack of support from superiors and co-workers [11,15], organizational changes [8,15,16], and downsizing [17] can lead to an environment prone to bullying and harassment. According to a recent systematic review by Brande, Baillien, Witte, Elst and Godderis [18], the most relevant work-related factors predicting workplace bullying are role conflict, workload, role ambiguity, job insecurity, and cognitive demands. Furthermore, the higher pace of change, increasing work intensity, and uncertainties about future employment may influence the level of stress, which provide fertile grounds for workplace violence, particularly bullying and harassment [19]. This hostile work environment can affect the health and well-being of the individual, increasing tension among co-workers and managers, reducing productivity, and lead to a rise in sickness-related absence and turnover rates [7].

According to Eurofound [19], employees in the public sector are more likely to have experienced bullying and harassment at work than those in the private sector, most probably due to comparatively higher levels of contact with the public. In line with that, the fifth European Working Conditions Survey showed that sectors with higher levels of contact with external clients, customers, patients, or students are prone to an increased risk of exposure to bullying and harassment [20]. Thus, it should not be of surprise that municipal employees are at a high risk of experiencing bullying and harassment at work [19,21,22,23,24]. According to Zapf’s [24] study, employees in the education sector, which often belongs to municipalities, were three times more likely to be bullied than employees in other sectors. In this light, and as the world is facing a deep economic crisis due to COVID-19, a longitudinal study as presented here, conducted in the wake of the economic crisis in 2008, among municipal employees within the education and care services is important, but rare. The study aims to improve the understanding of the development of lack of well-being, reflected in bullying and harassment during turbulent times, and increase the possibilities of proper actions being taken in such times, as requested by Hutchingson [2]. It is a four-wave panel study, asking whether bullying and/or harassment among those who work within education and care services, operated by Icelandic municipalities, changed during the economic downturn that began in 2008. Employing the job demand-control (JDC) model, which has rarely been used to analyze jobs in the educational sector, the relationship between factors such as downsizing, job security, job demands, and role conflict was analyzed, as a possible predictor of bullying at work during and after the economic crisis in Iceland.

### 1.1. Definitions and Frequency

Workplace bullying occurs when one or more employees, over a period, are repeatedly exposed to negative acts, torment, social exclusion, harassment, insults or offensive remarks, perpetrated by one or more individuals [25].

A study on U.S. workers by Rospenda, Richman, and Shannon [26] revealed that 63% of men and women had experienced one or more workplace harassment behaviors. Schat, Frone, and Kelloway [27] examined what they called workplace aggression in the U.S. workforce and found that 41% of the participants had experienced psychological aggression at work within the previous 12 months, and 13% on a weekly basis. European studies show that around 5% to 30% of the workforce in Europe have been victims of workplace bullying [16,28], while a nationwide American study by Lutgen-Sandvik et al. [29] revealed that 13% of their sample had experienced workplace bullying during the previous 12 months. According to Eurofound [19], approximately 6% of European employees reported having experienced some form of workplace violence, either physical or psychological, in the past 12 months. Levels of reported psychological violence (bullying or general harassment) are higher than physical violence, but bullying or harassment (12%) is more prevalent than sexual harassment (1%). Icelandic studies show that between 4% and 20% of employees have experienced bullying at work [30,31]. A recent study conducted by Snorradóttir et al. [31] revealed that 20% of employees had experienced bullying at work, 16% sexual harassment and 10% gender-based harassment. These numbers show the magnitude of the problem that bullying and harassment pose and the importance of creating a good healthy working environment for employees.

To differentiate between bullying and/or harassment and everyday conflicts in organizations, the persistence of the behavior is important. Most workplace bullying studies use a 12-month timeframe, since bullying usually involves repeated long-lasting conflicts. Einarsen and Skogstad [32] reported that the average exposure to bullying was 18 months, but in a study by Hoel and Cooper [33], 39% of the bullied employees reported that the bullying had lasted for more than two years. Vartia’s [34] study among Finnish municipal employees showed that 29% of the bullied employees had been bullied for two to five years and 30% had been exposed to it for more than five years. Keashly and Neuman [35] reported that more than a third of the bullied employees in a study of university faculty and staff had been bullied for more than three years. Therefore, a longitudinal panel study, as presented here, is essential. What our data adds to this knowledge is the time dimension, as it measures how changes in bullying and harassment occurred two, three, five, and seven years after the economic collapse in Iceland. Thus, we show changes in bullying and harassment among municipal employees working within the educational and care professions.

Different studies show conflicting results with respect to gender, age, and professions. Hoel and Cooper [33] and Hoel, Cooper, and Faragher [36] claimed that men and women are equally likely to be bullied. However, Zapf et al. [37] showed that 62.5% of victims of bullying were women, based on analyses of 53 studies on bullying. On the other hand, Eriksen and Einarsen [38] and Martino, Hoel, and Cooper [39] showed that gender minority is a risk factor. Thus, men are more likely to be bullied than women if the majority of employees are women, and vice versa. Furthermore, Vartia and Hyyti [40] found no gender difference in the number of employees being bullied among those who worked in a traditionally male-dominated environment, such as prisons. However, female prison workers have been subjects of sexual harassment significantly more often than their male colleagues [19]. Some studies have suggested that bullying and harassment in the workplace correlate with age, where younger employees are at a relatively greater risk of being bullied and experiencing more unwanted negative behaviors such as gender-based and sexual harassment than older employees (see, e.g., [19,39]). On the contrary, other studies have shown no such correlations [37].

### 1.2. Working Conditions in Times of Economic Crisis

According to Eurofound [19], conflict between employees, bullying, and violence at work increased in some European countries during the 2008 crisis. According to the report, rising job insecurity played an important intermediating role on lack of well-being in terms of stress and harassment. A common strategy when organizations face a difficult economic environment is to downsize [41,42,43]. It can be achieved through layoffs, or sometimes by less controversial managerial actions, such as restructuring, leaving vacated posts unfilled, and encouraging employees to retire voluntarily [41]. However, several studies have shown that organizational downsizing can have considerable negative consequences on the health and well-being of employees [44,45,46].

According to a literature review by Kulkarni [46], studies from 1984 to 2005 show major negative effects of downsizing on the remaining employees. Among these were psychosocial factors, such as creation of a climate of distrust, insecurity and demotivation, decreased employee satisfaction, low morale, poor commitment, increased absenteeism, greater workloads, a high level of stress, adverse social effects, and increased conflicts.

Adams and Flatau [47], Hearn and Parkin [48] Quinlan [49] and Landsbury, Johnson, and Broek [17] are among those who showed that downsizing is also a significant organizational factor that must be considered in relation to workplace bullying and harassment. According to Landsbury, Johnson and Borek [17] insecure job environments and workloads, caused by downsizing and organizational changes, are the most commonly cited work environment factors that increase the likelihood of bullying and harassment. Baillien and Witte [8] further showed a significant connection between organizational change and bullying, where role conflict, job insecurity and workload increased bullying, but social support from colleagues was shown to be related to bullying as a protective factor. Despite these studies, a deeper knowledge is needed about the connection between an economic crisis, which comes frequently, and often unexpectedly, and workplace bullying and harassment over a long time. This study will improve that knowledge by using longitudinal panel data to analyze bullying and harassment in times of an economic crisis, and also consider downsizing and other work organizational factors.

### 1.3. The Icelandic Setting and Research Questions

The economic crisis in Iceland, which struck in October 2008, is an illuminating case to study bullying and harassment in relation to job insecurity and other organizational issues in a turbulent time as it led to dramatic changes for many employees with increased job insecurity, unemployment and health problems as a consequence [50], as well as for employees of Icelandic municipalities [51].

Iceland, a rich modern, society belonging to the Nordic countries, was one of the first countries to go down in the wake of the Lehman Brothers’ collapse, which had a rippling effect on economies around the world [52]. The Icelandic crisis became particularly severe when all three national banks collapsed. The exchange rate fell dramatically overnight, followed by rapidly rising inflation, affecting almost every household, business and organization in Iceland. The unemployment rate rose from 2.1% to 7.4% among women and from 1.7% to 10.4% among men in the first years after the bank collapse [53]. Even though the Icelandic economy staged a relatively rapid recovery and indeed faster than anyone had predicted [54], the nation has been struggling in one way or other with the consequences of downsizing and the restructuring of labor market since then. That is a reality many nations in the world will be facing in the coming years, due to COVID-19.

In Iceland, similar to other Western countries, municipal employees have long enjoyed high levels of job security, where layoffs have been the exception. However, in the wake of the economic collapse, many municipalities reacted with cutbacks in services, but for many of them, that was not enough. As labor cost constitutes the largest single expenditure (around 60% of the total revenue), municipalities were forced to reduce their wage cost by reducing overtime, instituting hiring freezes, offering voluntary retirement, restructuring jobs, and laying off employees [55]. Municipality mayors have begun to step up in the media, pointing out similar issues related to today’s pandemic [56].

It was seen that in the wake of the economic collapse in 2008, employees’ health and well-being within education and care services in Iceland, operated by the municipalities, became worse [57]. The same applied for the banking sector five months after the collapse [50,58]. Based on that, we ask whether bullying and harassment have increased within the municipal education and care services, not only in the immediate aftermath of the collapse, but also whether the rate has returned to pre-collapse levels. More precisely, based on Kulkarni [46], we anticipated that job demands have increased in the wake of the economic collapse, and expect that this will lead to less role conflict and increased exposure to bullying and harassment at work. Hence, the research questions are as follows: (a) Has bullying and/or harassment increased between the time points of the study up to seven years after the crisis? (b) Did bullying and/or harassment change depending on downsizing? (c) Are quantitative job demands, role conflict and social support connected to bullying and/or harassment at work and, if so, how? By answering these questions and testing the hypotheses that (1) downsizing and; (2) increased job demands and role conflict lead to more bullying, but (3) the effects of these variables are mitigated through support by supervisors and coworkers, the aim is to further develop an understanding of the connection between these variables two, three, five, and seven years after the economic collapse. This knowledge is important when aiming to limit the negative effects on employees during downsizing during an economic crisis.

## 2. Materials and Methods

The data were collected from a longitudinal study called the Health and Well-Being of Employees of Municipalities in Iceland in Times of Economic Crisis. Participants covered about 50% of all employees of Icelandic municipalities. Most of the excluded municipalities in the study were very small or had fewer than 2000 inhabitants, except Reykjavik (capital of Iceland). About 80% of municipalities’ employees are female. The study was approved by the National Bioethics Committee of Iceland (VSN10-007).

### 2.1. Online Survey and Sample

A four-wave online survey was conducted among municipalities in Iceland, the first wave from February to April 2010 (the baseline study), the second from May to June 2011 (follow-up study 1), and the third from February to April 2013 (follow-up study 2), and the fourth from October to December 2015 (follow-up study 3). In each wave, three reminders were sent by e-mail. In order to monitor possible changes for each individual, the focus was on all employees in education (kindergarten teachers and primary school teachers) and care services (elderly care and care for people with disabilities), who responded to the survey at all four time points of the study. In 2010, the number of employees in the education and care services was 5717, and 4542 of them responded to the questionnaire (79%). Of the 4,542 baseline respondents, 4415 were still working 16 months later, at the time of follow-up study 1, and 3359 (76%) responded to the questionnaire a second time. Of the 3359 follow-up study 1 respondents, 3258 were still working 20 months later, during follow-up study 2. Of these, 2356 responded to the questionnaire a third time for follow-up study 2 (72%). Of the 2356 follow-up 2 respondents, 2261 were still working two and a half years later, during follow-up study 3. Of these, 1890 responded to the questionnaire a fourth time for follow-up study 3 (84% response rate or 33% of the original group of employees). Thus, the response rate through the years was between 72% and 84%, which is good. In this light, the data are strong, especially as this is not a sample but that all employees received the survey.

The majority participants were women (87.2%), and the average age was a little over 49 years for both women and men. The majority (65.2%), worked as primary school teachers, 25.3% as kindergarten teachers, and 9.4% worked in the care of elderly or disabled people. These percentages are representative of the population, but a slightly higher proportion of women than men participated in the survey. The number of participants vary slightly in the analyses, owing to some missing values of the predictor variables.

### 2.2. Survey Questions

The questionnaire devised for this study was based on two other questionnaires: the Icelandic version of the General Nordic Questionnaire for Psychological and Social Factors at Work (QPS Nordic) [59], and the questionnaire *Health and Well-being of the Icelandic Nation*, published by the Icelandic Public Health Institute, with additional questions about personnel reductions and internal reorganization. In this study, we used three questions about bullying and harassment in the current workplace, three questions about perpetrators of bullying and harassment, one question about downsizing, four questions about quantitative job demands, three questions about role conflict, one question about job security, three questions about support from supervisors, and two questions about support from co-workers. In addition, questions about gender, age, marital status, and occupation were used in the analysis.

The independent variables were gender (male or female), age (year of birth), marital status (single, married or cohabiting), and workplaces (kindergartens, primary schools, residences for older individuals and people with disabilities). Downsizing was measured with one question: “Have employees in your organization been laid off because of the economic collapse that occurred in October 2008?”—a yes/no question. Quantitative job demand was measured using the QPS Nordic four-item scale (“Is your workload irregular so that the work piles up?”, “Do you have to work overtime?”, “Is it necessary to work at a rapid pace?”, and “Do you have too much to do?”), with five response options (1 = very seldom or never to 5 = very often or always). This scale had adequate levels of internal consistency (Cronbach’s alpha = 0.77). Role conflict was measured using a three-question scale from QPS Nordic (“Do you have to do things that you feel should be done differently?”, “Are you given assignments without adequate resources to complete them?”, and “Do you receive incompatible requests from two or more people?”), with five response options (1 = very seldom or never to 5 = very often or always) (Cronbach’s alpha = 0.74). Job security was measured with the question: “Do you believe, if you want to, that you can keep your job at the municipality for the next 12 months?”—yes/no question. Support from superiors was measured using the QPS Nordic three-item scale (“If needed, can you get support and help with your work from your immediate superior/s?”, “If needed, is your immediate superior/s willing to listen to your work-related problems?”, “Are your work achievements appreciated by your nearest superior/s?”), with five response options (1 = very seldom or never to 5 = very often or always). (Cronbach’s alpha = 0.79). Support from co-workers was measured using the QPS Nordic two-item scale (“If needed, can you get support and help with your work from your co-workers?”, “If needed, are your co-workers willing to listen to your work-related problems”), with five response options (1 = very seldom or never to 5 = very often or always) (Cronbach’s alpha = 0.83).

Dependent variables were bullying and harassment, which were measured by the following three questions: (a) “Workplace bullying refers to hurtful and/or humiliating behavior toward the individual. The behavior is repeated and ongoing for some time, weeks, months, or years. Have you been bullied in your current workplace in the past 12 months?” (b) “Sexual harassment includes sexual activity, behavior, or comments directed at a person and against his or her consent or will. Have you experienced sexual harassment in your current workplace in the past 12 months?” (c) “Gender-based harassment is hurtful or humiliating conduct toward women or men because of their gender, such as ‘women are…’, ’men are…’, without being sexual. Have you suffered gender-based harassment in your current workplace in the past 12 months?” These were yes/no questions. We used these three questions also as one variable, describing bullying and/or harassment at work. This was done in such a way that if participants answered all three questions with *no*, they achieved the value 0 (79.9% of participants), but if they answered *yes* to any of these three questions, they achieved the value 1 (20.1% of participants). Only 0.4% of the participants answered all three questions with a *yes* for all time points of the study, 3.0% for two questions and 16.7% for one of the three questions. Hereafter, we refer to this new variable as bullying and/or harassment (binary variable, 0/1).

### 2.3. Statistical Processing

Exploratory data analysis was used to examine the relationship between the independent variables and the dependent variable with appropriate statistical tests—the chi-square test with continuity correction, Cochran’s Q test, and one-way repeated measures analysis of variance (ANOVA). A repeated-measures ANOVA was conducted to explore whether changes in the reported bullying and/or harassment were different between downsized workplaces and ones without, depending on quantitative job demands, role conflict, and support from superiors and co-workers. Generalized estimating equations (GEEs) were used to evaluate the impact of downsizing, quantitative job demands, role conflict, job security, support from superiors and support from co-workers on bullying and/or harassment over time, and at specific time points. The GEE method has several advantages for analyzing longitudinal data; for example, it uses all available data points, provides a method for handling the correlated nature of repeated measurements, and accounts for the pattern of change over time [60]. A binomial distribution with logit link function was used to evaluate the dichotomous dependent variable (bullying and/or harassment). We selected a first-order exchangeable correlation structure. In model 1, the main effects of downsizing, study time point, quantitative job demands, and role conflict were examined. In model 2, support from superiors and co-workers was added to model 1. To test our hypotheses that the effects of downsizing depend on both role conflict and support from superiors and co-workers, the interaction between downsizing and quantitative job demands, interaction between downsizing and role conflict, and interaction between downsizing, role conflict, and support from superiors and co-workers were added to model 3. We obtained the odds ratio (OR) and associated the *p* value for each predictor in the GEE models. SPSS 21.0 was used to conduct all data analyses.

## 3. Results

### 3.1. Bullying and Harassment

As shown in Table 1, the proportion of bullied employees in kindergartens, primary schools and residences for older individuals or people with disabilities was 8% in 2010, and rose to more than 13% in 2011, and nearly 20% in 2013 and 2015. The most common perpetrators of bullying were co-workers, at all time points (69%, 57%, 54%, and 73%, respectively, not shown in the table), and the second most common perpetrators were the managers or supervisors of the respondents (33%, 40%, 44%, and 33%, respectively, not shown in the table).

A much lower proportion of employees had experienced gender-based and sexual harassment than bullying or around 3 to 5% in 2010, and changes between time points were much smaller than in the case of bullying. The proportion of reported sexual harassment rose by 1.7% between 2010 and 2011, stayed the same in 2013, and went down again by 1.4% between 2013 and 2015. Gender-based harassment showed a similar trend with an increase of 1.8% between 2010 and 2011, a very slight increase between 2011 and 2013 and, finally, a reduction by 2% between 2013, and 2015, ending with a lower rate of employees reporting experience of gender-based harassment in 2015 than in 2010 (see Table 1). The perpetrators in the gender-based and sexual harassment among employees in kindergartens and primary schools were their co-workers or managers/supervisors; however, perpetrators were residents among those who worked in residences for older individuals or people with disabilities. The percentage of employees who had experienced sexual harassment at all four time points was 0.8%, and 1.3% reported having experienced gender-based harassment for the all time period.

Table 2 shows that the proportion of employees in kindergartens, primary schools and residences for older individuals or people with disabilities who had been exposed to bullying and/or harassment increased significantly over the course of the time of study, rose from 13.5% in 2010 to 25.8% in 2013 but was 22.6% in 2015. No gender differences were found in exposure to bullying and other types of harassment at work at any of the four time points (*p* > 0.05).

Bullying and/or harassment increased between all time points, for both women and men, all age groups, single participants, married or cohabiting, and all workplaces groups (see Table 2). The gender difference was not significant, but single participants experienced more bullying and/or harassment than cohabiting or married participants, at all time points.

**Figure 1:**
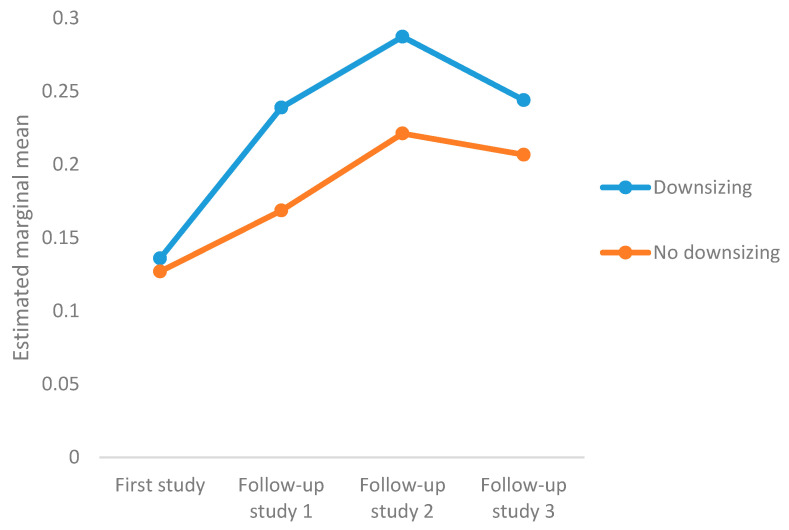
Changes in bullying and/or harassment over time in workplaces that underwent downsizing and workplaces that did not. Note, covariates appearing in the model are evaluated at the following values: quantitative job demand = 3.2, role conflict = 2.3, support from superiors and co-workers = 4.0.

The proportion of those who had experienced bullying and/or harassment also varied by workplaces. A higher proportion of employees working in residences for older individuals and people with disabilities reported having experienced bullying and/or harassment than employees working in kindergartens and primary schools, both in the first and follow-up studies. Furthermore, a higher proportion of employees working in workplaces where downsizing had occurred experienced bullying and/or harassment than those working in workplaces with no downsizing. See Figure 1.

### 3.2. Working Condition in Times of Economic Crisis

In 2010, about 27% of employees in kindergartens, primary schools and residences for older individuals or people with disabilities reported that there had been layoffs at their workplaces because of the economic collapse. The proportion rose to 45% in 2011, to 51% in 2013 and to 52% in 2015, seven years after the bank collapse. The increase in reported downsizing was significant between the time points of the study [χ^2^_(2, 1890)_ = 1088.6; *p* ≤ 0.05]. However, even though layoffs occurred and increased between the study points, 98% of the employees reported that they believed they would keep their job at the municipality for the next 12 months, at all points. In other words, perceptions of job security stayed the same during the three time points of the study (see Table 3).

Table 3 outlines descriptive statistics for quantitative job demand, role conflict, and support from superiors and co-workers in 2010, 2011, 2013 and 2015. In 2010, the scale mean (scale 1–5) for quantitative job demand was 2.8 (standard deviation (Sd) = 0.8), and rose to 2.9 (Sd = 0.8) in 2011, 3.0 (Sd = 0.9) in 2013, and 3.2 (Sd = 0.9) in 2015. There was a significant increase in quantitative job demands over time [*F* (2, 2347) = 111.4, *p* ≤ 0.05]. Furthermore, quantitative job demands were higher in downsized workplaces at all four time points [2010: mean 2.9 (Sd = 0.8) vs. 2.7 (Sd = 0.8); *t*_(1888)_ = −5.88, *p* ≤ 0.05; 2011: mean 3.0 (Sd = 0.8) vs. 2.7 (Sd = 0.8); *t*_(1888)_ = −8.05, *p* ≤ 0.05. 2013: mean 3.2 (Sd = 0.8) vs. 2.8 (Sd = 0.8); *t*_(1888)_ = −9.46, *p* ≤ 0.05. 2015: mean 3.3 (Sd = 0.8) vs. 3.1 (Sd = 0.8); *t*_(1888)_ = −5.27, *p* ≤ 0.05].

Role conflict increased slightly between the first three time points 2010 and 2013. but remained the same for 2013 and 2015 [*F* (2, 2342) = 44.0, *p* ≤ 0.05]. In workplaces were employees did not report layoffs, they experienced less role conflict than where participants reported layoffs [2010: *t*_(1886)_ = 5.28, *p* ≤ 0.05; 2011: *t*_(1885)_ = 4.72, *p* ≤ 0.05; 2013: *t*_(1883)_ = 6.20, *p* ≤ 0.05. 2015; *t*_(1888)_ = 4.67, *p* ≤ 0.05]. The scale mean in workplaces where employees did not report layoffs was 4.1 (Sd = 0.8) in 2010, 4.1 (Sd = 0.7) in 2011, 4.1 (Sd = 0.9) in 2013, and 4.0 (Sd = 0.9) in 2015. However, it was 3.9 (Sd = 0.9) in 2010 in workplaces where employees reported layoffs, 3.9 (Sd = 0.9) in 2011, 3.8 (Sd = 0.9) in 2013, and 3.8 (Sd = 0.9) in 2015.

Both support from superiors and co-workers decreased between all four time points of the study (*p* ≤ 0.05). The means and standard deviations are presented in Table 3. Employees in workplaces with no downsizing reported higher support from their superiors than where downsizing had occurred at all four time points [2010: mean 4.2 (Sd = 0.8) vs. 4.1 (Sd = 0.8); *t*_(1886)_ = 4.26, *p* ≤ 0.05; 2011: mean 4.2 (Sd = 0.7) vs. 4.1 (Sd = 0.8); *t*_(1885)_ = 3.84, *p* ≤ 0.05; 2013: mean 4.2 (Sd = 0.8) vs. 4.0 (Sd = 0.8); *t*_(1882)_ = 4.24, *p* ≤ 0.05; 2015: mean 4.1 (Sd = 0.8) vs. 4.0 (Sd = 0.8); *t*_(1888)_ = 2.76, *p* ≤ 0.05]. The same pattern was observed for support from co-workers, which lowered in workplaces where employees reported layoffs than those without [2010: mean 4.1 (Sd = 0.8) vs. 4.2 (Sd = 0.8); *t*_(1886)_ = 4.26, *p* ≤ 0.05; 2011: mean 4.1 (Sd = 0.8) vs. 4.2 (Sd = 0.7); *t*_(1885)_ = 3.84, *p* ≤ 0.05; 2013: mean 4.0 (Sd = 0.8) vs. 4.2 (Sd = 0.8); *t*_(1882)_ = 4.24, *p* ≤ 0.05; 2015: mean 4.0 (Sd = 0.8) vs. 4.1 (Sd = 0.8); *t*_(1888)_ = 2.76, *p* ≤ 0.05].

### 3.3. Working Condition and Bullying and/or Harassment at Work

Table 4 outlines the results of the GEE analysis of main effects (Model 1 and Model 2) and main effects and interactions (Model 3) predicting the likelihood of bullying and/or harassment over time. Job security stayed the same and was not significantly associated with bullying and/or harassment and, therefore, excluded from the models.

In Model 1, downsizing, time point of the study, quantitative job demand, and role conflict were all significantly associated with bullying and/or harassment over time, indicating that employees in workplaces where downsizing occurred (OR = 1.19), in workplaces with higher quantitative job demand (OR = 1.31), and higher role conflict (OR = 1.17) were more likely to report experiences of bullying and/or harassment at work over time (controlling for other factors in the model). Even after adding support from superiors and co-workers to the model, downsizing (OR = 1.8), quantitative job demands (OR = 1.30) and role conflict (OR = 1.12) remained significant factors, but support from superiors and co-workers showed mitigating effects (OR = 0.8) on bullying and/or harassment over time (controlling for other factors in the models).

The goodness of fit of the models in Table 4 improved with adding support from superiors and co-workers, interactions between downsizing, quantitative job demands and role conflict (Quasilikelihood under the Independence Model Criterion (QIC) and Corrected Quasi-likelihood under the Independence Model Criterion (QICC) become smaller. A significant interaction between downsizing and quantitative job demands shows that the influence of downsizing was less in workplaces where quantitative job demands were lower. Model 3 also reveals a significant three-way interaction between downsizing, role conflict, and support from superiors and co-workers. The results indicate that the influence of downsizing and role conflict were buffered by support from superiors and co-workers (OR = 0.92).

## 4. Discussion and Conclusions

In this study, possible changes in workplace bullying and harassment was explored among employees working within the education and care services operated by 17 municipalities in Iceland, in the wake of the 2008 economic collapse. The focus was on those who remained at work two, three, five and seven years after the collapse.

In short, the results show that bullying and/or harassment increased between all the time points of the study. The proportion was lowest in 2010 (13%) but doubled in 2013 (26%). Employees in workplaces with downsizing were more likely to report experience of bullying and/or harassment at all time points of the study. The differences were highest in 2013 when 9% more employees reported being exposed to bullying and/or harassed in workplaces with downsizing than those without. These results show the importance of downsizing as a factor in working environment influencing bullying, and harassment as Landsbury et al. [17] pointed out in their research. It is important to emphasize that this relationship is evident, even if employees in our study do not experience job insecurity as in the case with most of the studies cited.

It is noteworthy that managers use downsizing to cut costs in times of economic crisis, even though it can lead to a hostile working environment. The results further show that employees in workplaces with higher quantitative job demands and more role conflict were more likely to experience bullying and/or harassment than employees in workplaces where quantitative job demands remained low over time. Importantly the influence of downsizing and role conflict on bullying and/or harassment were buffered by support from superiors and co-workers. These results are in line with Baillien and Witte’s [8] study, which stated that role conflict and workload increased the likelihood of bullying, but social support was a protective factor. This tells us that managers and those who are responsible for well-being at work should always emphasize social support, not the least in turbulent times, and in workplaces where the psychosocial environment is strenuous.

It was not necessarily unexpected that quantitative job demands increased in the wake of the economic crisis due to cuts in funding to the municipalities. Unsurprisingly, the employees also experienced less control over work. However, this relationship has been more researched in monotonous and repetitive work than in education and care services. This is in line with the job demand and control model, introduced by Karasek [61] and Karasek and Theorell [62] in the field of job stress and strain.

Surprisingly, there was no connection between job security and bullying and/or harassment, although other studies have confirmed such connections (see [47,48,49]). Of course, municipalities have been considered workplaces with a high job security, due to legal roles related to education and care. Nevertheless, the employees in our study faced a cut-down in manpower after the bank crisis in 2008.

The literature review showed that results regarding gender and age differences in bullying and harassment are contradictory. As Eriksen and Einarsen [38] and Martion, Hoel and Cooper [39] showed that gender minority is a risk factor, this research should possibly have shown that men were at greater risk. However, this study showed no significant gender differences. Both women and men and employees in all age groups experienced increased job demands, more role conflict, and downsizing over the studied period. But being married or cohabiting was a protective factor in all time points of the study. It cannot be explained, based on this data, why this is a protective factor, but this is probably because being able to share a life with another person makes it easier for individuals to think of something else other than work while at home. It is a concern that the situation deteriorated by all measures up to the year 2013—that is five years after the economic crisis started, and it is interesting to see that the situation improved somewhat in 2015. This indicates that the situation has started to improve again. Nevertheless, in cases, the situation was worse in 2015 than in the first wave in 2010. In this light, what happens in 2020 due to COVID-19 is a matter of worry as, clearly, the financial situation of the municipalities has worsened sharply, due to the pandemic.

The strength of the study is that it is based on recent longitudinal panel data, where employees were followed two, three, five and seven years after the economic collapse in Iceland. In addition, the response rate was good (72–84%), covering about 50% of those employed by Icelandic municipalities. The fact that we did not work with a sample, as all the employees received the survey, also makes the data strong.

Despite the strength of the study, several limitations can be addressed. Firstly, based on this data, we cannot draw any conclusions about the cost of bullying and harassment. As various workplaces are governed by economic factors rather than those related to people’s well-being, we hope that future research will evaluate this. Secondly, even though it is important to divide the workplaces into two categories, based on whether they had been downsized, it is not possible to classify them according to the magnitude of downsizing, as we did not have such data. Thirdly, it can be seen as a weakness to use only one question to measure bullying and two questions to measure harassment. In this context, however, we argue that we are not analyzing the nature of bullying or harassment in the workplaces under scrutiny, but rather whether the employees have/had been exposed to this behavior in general, and how it relates to the time factor and the organizational issues under examination. Fourth, even though we have longitudinal data, we cannot distinguish between the effects of organizational factors and possible spreading of effects, good or bad, from outside the workplace. Although we found convincing connections between increased job demands, role conflict, lack of social support, and bullying and harassment, at all time points of the study, it is difficult to establish with certainty the extent to which this can be traced directly to what happens within the workplace, or whether the situation outside the workplace has an influence as well.

The situation for municipal employees differed after the economic collapse in 2008, compared to employees in many other sectors, such as banking, which has been discussed in other studies [50,58]. In contrast to the extraordinary mass layoffs among employees in the banking and construction sectors [58], the downsizing of the workforce in municipalities was primarily carried out by a reduction in overtime and substitute workers, implementation of a hiring freeze, offering voluntary retirement, and restructuring jobs. The “silent” downsizing of the workforce combined with people’s notion that job security in the public sector surpassed that of private industry, resulted in little discussion, understanding, or even sympathy among the public at large, for the situation of municipal employees. Despite the cutbacks in their operations, municipalities were, however, still legally obligated to provide welfare services for all residents, including education. Hence, the combination of a reduction of the workforce, having fewer resources available to perform standard operations, while simultaneously being obliged to maintain the same level of services as before, led to a considerably increased strain on municipal employees. Nevertheless, we do not know for sure if the increase in bullying and harassment only had to do with poorer work conditions, or to what extent it also had to do with deteriorating personal circumstances outside the workplace, and/or increased awareness of the problem. Despite this, we view the results as presenting a good understanding of changes in bullying two to seven years after the economic collapse.

Hutchinson [2] argued that as theorizations and policy definitions tend to overlook the significance of organizational factors of bullying, the capacity to prevent or resolve the problem is limited. Our results support studies showing that organizational factors are vital when preventing workplace bullying. Therefore, we urge those who are responsible for prevention and solution-oriented actions in workplaces to strongly focus on these aspects.

Theoretically the results show that the JDC model can be used to shed light on the working conditions of employees within education and care services. In addition, “silent downsizing”, that is downsizing where no lay-offs among employees have occurred, can trigger the feeling of increased job demands, and inappropriate behavior, such as bullying and harassment, even though job insecurity does not increase.

In addition to the theoretical impact of how to use the JDC model in education and care services, one of the main impacts lies in the time dimension used in the study, showing how changes in bullying and harassment changed over time. It adds important elements to existing knowledge, as it is socially and politically vital to understand how negative consequences of the economic crisis in 2008 manifested in changes in bullying and harassment for up to seven years after the crisis. Since the effects may prevail for several years, the study demonstrates the importance of preserving the work organization and workplace behavior of all employees. As social support, particularly managerial support, proved to be a buffer against other assessed factors in our study, it is important to find ways to strengthen managers in this role. As economic crises tend to occur periodically, and a deep crisis is imminent due to COVID-19, organizations should be aware of this and take active measures to ensure that the workplace is a safe and secure place for every employee. This knowledge is also of importance for those who work with policies for the reduction of bullying and harassment in their services. Even though our study is on employees working within education and care services operated by Icelandic municipalities, we believe that the main results touch upon fundamental elements, such as well-being at work, and can be applied to other and different workplaces as well.

## Figures and Tables

**Table 1:** Proportion having experienced bullying, sexual harassment and gender-based harassment by gender in 2010, 2011, 2013, and 2015.

	First Study (2010)	Follow-Up Study 1 (2011)	Follow-Up Study 2 (2013)	Follow-Up Study 3 (2015)	Cochran’s Q Test χ^2^	% in All Four Time Points
Bullying	7.8%	13.0%	19.5%	19.6%	270.5 ***	4.1%
Women	8.0%	13.0%	19.2%	19.5%	227.4 ***	4.4%
Men	6.2%	12.9%	21.2%	19.9%	43.7 ***	2.5%
Sexual harassment	3.4%	5.1%	5.1%	3.7%	21.3 ***	0.8%
Women	3.5%	5.3%	5.4%	3.7%	21.3 ***	0.7%
Men	2.5%	3.7%	3.3%	3.7%	1.8	1.7%
Gender-based harassment	4.5%	6.3%	6.7%	3.7%	49.8 ***	1.3%
Women	4.2%	6.1%	6.6%	3.5%	46.6 ***	1.2%
Men	6.2%	7.9%	7.1%	5.0%	4.7	2.1%
Bullying and/or harassment	13.3%	20.6%	25.7%	22.6%	181.7 ***	5.9%
Women	13.5%	20.8%	25.8%	22.5%	155.9 ***	6.1%
Men	12.0%	19.5%	25.3%	23.2%	26.1 ***	4.1%

Note. *** *p* ≤ 0.001,

**Table 2:** Frequency of bullying and/or harassment in 2010, 2011, 2013, and 2015 by gender, age, marital status and workplaces.

	Bullied and/or Harassed in 2010 *n* (%)	Bullied and/or Harassed in 2011 *n* (%)	Bullied and/or Harassed in 2013 *n* (%)	Bullied and/or harassed in 2015 *n* (%)	Cochran’s Q Test χ^2^
Total	251 (13%)	390 (21%)	486 (26%)	426 (23%)	181.7 ***
Gender					
Female	222 (14%)	343 (21%)	425 (26%)	370 (23%)	155.9 ***
Male	29 (12%)	47 (20%)	61 (25%)	56 (23%)	26.1 ***
Age (years)					
<30	10 (14%)	15 (21%)	17 (24%)	14 (20%)	4.3
31–40	54 (13%)	81 (19%)	108 (26%)	106 (25%)	55.8 ***
41–50	87 (13%)	132 (20%)	160 (24%)	152 (23%)	55.5 ***
51–60	73 (13%)	117 (21%)	151 (27%)	119 (22%)	64.3 ***
>60	27 (14%)	44 (23%)	49 (25%)	33 (17%)	17.6 ***
Marital status					
Single	56 (18%)	77 (25%)	95 (31%)	83 (27%)	25.1 ***
Married or cohabiting	195 (12%)	312 (20%)	390 (25%)	343 (22%)	156.8 ***
Workplaces					
Primary schools	156 (13%)	255 (21%)	309 (25%)	278 (23%)	121.0 ***
Kindergartens	64 (13%)	86 (18%)	113 (24%)	97 (20%)	36.8 ***
Residences for older individuals or people with disabilities	31 (18%)	49 (28%)	64 (36%)	51 (29%)	25.9 ***
Downsizing					
Workplaces with downsizing	70 (14%)	216 (25%)	290 (30%)	252 (26%)	134.3 ***
Workplaces with no downsizing	181 (13%)	174 (17%)	196 (21%)	174 (19%)	54.3 ***

Note. *** *p* ≤ 0.001, Marital status: 2010: (χ^2^_(1, 1890)_ = 7.1; *p* ≤ 0.05), 2011: (χ^2^_(1, 1890)_ = 9.6 *p* ≤ 0.05); 2013: (χ^2^_(1, 1890)_ = 7.9; *p* ≤ 0.05), 2015: (χ^2^_(1, 1883)_ = 3.9; *p* ≤ 0.05). Workplaces: 2011: χ^2^_(2, 1890)_ = 7.5; *p* ≤ 0.05; 2013: χ^2^_(2, 1890)_ = 11.6; *p* ≤ 0.05; 2015: χ^2^_(2, 1884)_ = 6.0; *p* ≤ 0.05). Downsizing: 2011: (χ^2^_(1, 1890)_ = 19.9 *p* ≤ 0.05); 2013: (χ^2^_(1, 1890)_ = 17.8; *p* ≤ 0.05), 2015: (χ^2^_(1, 1884)_ = 12.2; *p* ≤ 0.05).

**Table 3:** Descriptive statistics for downsizing, job security, quantitative job demand, role conflict, support from superior and support from co-workers in 2010, 2011, 2013 and 2015.

	First Study (2010)		Follow-Up Study 1 (2011)		Follow-Up Study 2 (2013)		Follow-Up Study 3 (2015)		Cochran’s Q Test
	N	%	N	%	N	%	N	%	χ^2^
Downsizing	506	26.8	855	45.2	970	51.3	977	51.7	1088.6 ***
Job security	1849	97.9	1853	98.2	1855	98.3	1866	98.7	3.0
	**Scale Mean**	**Scale Sd**	**Scale Mean**	**Scale Sd**	**Scale Mean**	**Scale Sd**	**Scale Mean**	**Scale Sd**	**One-Way Repeated Measures ANOVA *F***
Quantitative job demand (1)	2.8	0.8	2.9	0.8	3.0	0.9	3.2	0.8	157.8 ***
Role conflict (2)	2.1	0.8	2.2	0.8	2.3	0.8	2.3	0.8	49.5 ***
Support from superiors (3)	4.1	0.9	4.0	0.9	3.9	0.9	3.9	0.9	28.7 ***
Support from co-workers (4)	4.2	0.8	4.1	0.8	4.1	0.8	4.0	0.8	17.8 ***

Note. *** *p* ≤ 0.001, (1) Wilks’ Lambda = 0.91, *F* (2, 2347) = 111.4, *p* ≤ 0.05, multivariate partial eta squared = 0.09. (2) Wilks’ Lambda = 0.98, *F* (2, 2342) = 44.0, *p* ≤ 0.05, multivariate partial eta squared = 0.02. (3) Wilks’ Lambda = 0.98, *F* (2, 2331) = 35.5, *p* ≤ 0.05, multivariate partial eta squared = 0.03. (4) Wilks’ Lambda = 0.98, *F* (2, 2337) = 25.5, *p* ≤ 0.05, multivariate partial eta squared = 0.02.

**Table 4:** Generalized estimating equation analyses predicting likelihood of bullying and/or harassment.

	Model 1	Model 2	Model 3
	OR	OR	OR
Intercept	0.047 ***	0.128 ***	0.063 ***
Downsizing	1.194 **	1.177 ***	0.853
Follow up study 1	1.593 ***	1.589 ***	1.600 ***
Follow up study 2	2.010 ***	2.003 ***	2.011 ***
Follow up study 3	1.602 ***	1.612 ***	1.624 ***
Quantitative job demands	1.308 ***	1.295 ***	1.172 ***
Role conflict	1.170 ***	1.115 **	1.725 **
Support from superior and co-workers		0.804 ***	1.001
Downsizing * Quantitative job demands			1.280 ***
Downsizing * Role conflict			0.841
Downsizing * Role conflict * Support from superior and co-workers			0.916 *
Goodness of fit (QIC)	6767.57	6681.06	6657.06
Goodness of fit (QICC)	6768.05	6682.17	6661.36

Note. *** *p* ≤ 0.001, ** *p* ≤ 0.01, * *p* ≤ 0.05.
